# Paternal postnatal depression in Japan: an investigation of correlated factors including relationship with a partner

**DOI:** 10.1186/s12884-015-0552-x

**Published:** 2015-05-31

**Authors:** Akiko Nishimura, Yuichi Fujita, Mayumi Katsuta, Aya Ishihara, Kazutomo Ohashi

**Affiliations:** Department of Nursing, School of Nursing, Hyogo University of Health Sciences, 1-3-6 Minatojima, Chuo-ku, Kobe, 650-8530 Japan; Department of Nursing, Japan Red Cross Hiroshima College of Nursing, 1-2 Ajinadaihigashi, Hatsukaichi, 738-0052 Hiroshima Japan; Department of Children and Women’s Health, Osaka University Graduate School of Medicine, 1-7 Yamadaoka, Suita, 565-0871 Osaka Japan

**Keywords:** Paternal postnatal depression, Father, Relevant factors, Relationship with a partner

## Abstract

**Background:**

A negative effect of paternal depression on child development has been revealed in several previous studies. The aims of this study were to examine the prevalence and relevant factors associated with paternal postnatal depression at four months postpartum, including age, part-time work or unemployment, experience of visiting a medical institution due to a mental health problem, economic anxiety, unexpected pregnancy, pregnancy with infertility treatment, first child, partner’s depression, and lower marital relationship satisfaction.

**Methods:**

We distributed 2032 self-report questionnaires to couples (one mother and one father) with a 4-month old infant between January and April 2013. Data from 807 couples (39.7 %) were analyzed. Depressive symptoms were measured with the Edinburgh Postnatal Depression Scale (EPDS). In order to clarify the factors related with paternal depression, a logistic regression analysis was conducted.

**Results:**

One hundred and ten fathers (13.6 %) and 83 mothers (10.3 %) were depressed. According to the logistic regression analysis, paternal depression was positively associated with partner’s depression (adjusted odds ratio (AOR) 1.91, 95 % confidence interval (CI) 1.05–3.47), and negatively with marital relationship satisfaction (AOR 0.83, 95 % CI 0.77–0.89). History of infertility treatment (AOR 2.37, 95 % CI 1.32–4.24), experience of visiting a medical institution due to a mental health problem (AOR 4.56, 95 % CI 2.06–10.08), and economic anxiety (AOR 2.15, 95 % CI 1.34–3.45) were also correlated with paternal depression.

**Conclusions:**

This study showed that the prevalence of paternal depression at four months after childbirth was 13.6 % in Japan. The presence of partner’s depression and low marital relationship satisfaction were significantly correlated with paternal postpartum depression, suggesting that health professionals need to pay attention to the mental status of both fathers and mothers, and to their relationship.

## Background

The influence of paternal depression on child development has been revealed in several large-scale longitudinal studies [1–4]. In the UK, if fathers experience depression by 8 weeks after childbirth, the risk of their child developing behavioral and emotional problems at 3.5 years old is doubled, and the risk of mental illness at 7 years old increases 1.7-fold [1, 2]. A large-scale longitudinal study in the US targeting 20,000 parents and children revealed that paternal depression predicted negative effects on child development [3]. Additionally, in Australia, Fletcher et al. reported that early paternal depression was a significant predictor of a child’s behavioral difficulties and low developmental outcomes from a large-scale study [4]. As described above, it seems evident that paternal depression is harmful for child development.

Paternal depression has been reported to correlate with an unwillingness to participate in child-rearing. Compared to non-depressed fathers, depressed fathers have a significantly lower engagement in positive enrichment activity with the child [5]. Furthermore, depressed fathers are more likely to use physical discipline with children who are less than 12 months old than non-depressed fathers [6]. Therefore, the identification of factors relevant to paternal postnatal depression is important for its prevention and early detection.

Goodman revealed partner’s depression as a relevant factor in paternal postnatal depression [7], and similar results were reported in subsequent systemic reviews [8, 9]. In addition, paternal postnatal depression was reported to correlate with a poor marital relationship [10, 11]. Mothers’ child-rearing difficulties and dissatisfaction in the marital relationship were associated with fathers’ postnatal depression 1 to 6 months after childbirth [12]. It appears that partners’ depression and the quality of the relationship with partners might be related to paternal postnatal depression.

There have been only three studies of paternal depression in Japan [13–15]. The prevalences of paternal postpartum depression one month after childbirth were reported to be 11.6 % [13] and 13.6 % [14]. The prevalence of paternal depression increased from 13.6 % during partners’ pregnancy to 16.3 % at 6 months and 19.4 % at 12 months after childbirth [15]. In a meta-analysis by Paulson et al., the prevalence of paternal depression from early pregnancy to 1 year after childbirth was 10.4 %, with the lowest prevalence being 7.7 % from childbirth to 3 months postpartum and the highest being 25.6 % from 3–6 months after childbirth [8]. Since April 2009, home-visit support for all families with infants was initiated according to the Child Welfare Act in Japan. On verifying the results of death cases among abused Japanese children in 2012, the abuse cases were mostly infants up to 4 months old [16], and the correlation between abuse and postpartum depression should be analyzed. However, there are no data on depression symptoms of fathers in Japan at 4 months postpartum. Accordingly, this study was the first to include fathers at 4 months after childbirth, when the prevalence of paternal depression is likely the highest as described by Paulson et al.

The aims of the present study were to examine 1) the prevalence of postnatal depression among Japanese fathers at four months postpartum, and 2) the factors associated with paternal postnatal depression, including age, part-time work or unemployment, experience of visiting a medical institution due to a mental health problem, economic anxiety, unexpected pregnancy, pregnancy with infertility treatment, first child, partner’s depression, and lower marital relationship satisfaction.

## Methods

### Participants

We distributed 2032 self-report questionnaires to couples (one mother and one father) with a 4-month old infant between January and April 2013. Almost a half of Japanese mothers traditionally stay at their parents’ home before and for one month after childbirth, and most of these mothers return to their home to live together with the father and their newborn from one month postpartum. A 1-month examination for all postpartum mothers and infants is routinely performed at hospitals or clinics where mothers delivered, and maternal and child health support services including a 4-month infant examination are provided for all families by municipal governments, and the participation rate for the 4-month infant examination was 95.5 % in 2012 [17]. The Ministry of Health, Labour and Welfare, Japan, recommend midwives and public health nurses to screen the depressive status with the EPDS at 1-month postpartum, and to initiate appropriate procedures for positive cases. However, the rate of implementing EPDS screening was 11.5 % in 2013 [18].

After receiving notification from municipal governments, parents bring their infants to health centers in their residential areas and undergo population screening and individual consultation. Along with notification of the 4-month infant health examination, a participation information sheet, a written request to participate in the study, and questionnaires for mothers and fathers were sent to all mothers four months after childbirth in four wards of Kobe City, Japan. Kobe city is located in western Japan and has a population of about one million five hundred thousand living in nine wards. Per capita citizen’s income was 2,900,000 yen in 2013 (per capita national income 3,500,00 yen in 2012). The four wards cover about half the population of Kobe city and are representative of the east, west, north and central areas of Kobe city.

The participation information sheet documented the purpose and methods of this study, privacy protection, absence of disadvantages related to the presence or absence of study cooperation, and freedom of discontinuation. Each questionnaire was given a random number which was not associated with the particular person. The questionnaires were to be completed individually by each mother and father without any discussion between the two, and sealed in an envelope at home. At the 4-month infant health examination, mothers and/or fathers brought the sealed envelopes and posted them in a collection box in the examination room. The researcher recovered and stored them in a safe located in Hyogo University of Health Sciences. We considered that the submission of the questionnaire was equivalent to consent to research participation, and did not separately obtain written informed consent from each subject in the study. When participants felt mental stress during or after the investigation, they could directly contact the researcher (Nishimura) by e-mail or mobile phone, or visit the obstetrician (Ohashi) to consult about their mental condition.

The study was conducted with the approval of the Hyogo University of Health Sciences Ethical Review Committee (number 12040).

### Measurements

#### Outcome variables

The Edinburgh Postnatal Depression Scale (EPDS) is a maternal postnatal depression screening scale developed by Cox et al. [19]. It consists of ten short statements: eight addressing depressive symptoms (e.g. sadness, self-blame) and two inquiring about anxiety symptoms (e.g., feeling worried or anxious and feeling scared or panicky). The fathers and mothers underlined which of the four possible responses was closest to how they had been feeling during the past week. A total score from the 10 items is calculated and used for evaluating depression. The EPDS has adequate reliability and validity based on studies conducted worldwide, and the reliability and validity of the Japanese version of the EPDS was verified by Okano et al.; a cut-off point of 8/9 showed a sensitivity of 75 % and specificity of 93 % [20].

The reliability and validity of the EPDS has been verified among men [12, 21, 22]. We performed an analysis of Japanese men’s EPDS cut-off points and found that a score of 8 points or higher indicates ‘depressed’ with a sensitivity of 81.8 % and specificity of 94.1 % [13]. Cronbach’s alpha of the EPDS for the current sample of fathers was 0.81.

#### Explanatory variables

We obtained the data by participants’ self-reporting on a questionnaire. Their questions and response options are summarized in Table 1. Demographic characteristics such as age, educational level, and employment status were investigated. Regarding health status, the presence or absence of current illness and use of psychotropic drugs (anti-depressants, anti-anxiety agents, mood-stabilizing agents, and sleeping pills) were asked. Regarding psychological factors, experience of visiting a medical institution due to a mental health problem, life events in the previous year, and the presence or absence of economic anxiety were addressed. We did not determine the type of medical institution or the history of hospitalization. Economic anxiety means anxiety about the family budget. Information on pregnancy, childbirth, and engaging with the child was obtained by asking about the timing of pregnancy, expected or unexpected pregnancy, the presence or absence of infertility treatment, the couple’s first child or not, single or multiple pregnancy, gestational age, health status of child, and living with their partner or apart.

**Table 1:** List of questions and answers in the questionnaire

Question items	Response options
Demographic characteristics	
Age	Age at last birthday (years)
Educational level	University graduate or higher. No = 0, Yes = 1
Employment status	Full-time = 0, part-time or unemployed = 1
Health status	
Current illness	Father was asked whether he has any illness including psychiatric disorders.
Psychotropic drugs	Father was asked whether he takes any drugs for mental illness. Choices were as follows; antidepressants, anti-anxiety agents, mood-stabilizing agents, and sleeping pills
Psychological factors	
Experience of mental problems	Father has experience of visiting a medical institution due to a mental health problem. No = 0, Yes = 1
Life events	Whether a family member or close friend died or suffered a serious injury or accident in the previous 1 year. No events = 0, one or more events = 1
Economic anxiety	Father has anxiety about the family budget. No = 0, Yes = 1
Pregnancy, childbirth, and engaging with the child	
Timing of pregnancy	Pregnancy after marriage = 0, Pregnancy before marriage = 1
Planning of pregnancy	Unexpected pregnancy. No = 0, Yes = 1,
Conception method	Pregnancy with infertility treatment. No = 0, Yes = 1
Order of birth	Second child or more = 0, First child = 1
Number of children in this pregnancy^a^	Single birth = 0, Multiple birth = 1
Gestational age^a^	Gestational week at delivery
Health status of child^a^	Child has a health problem. No = 0, Yes = 1
Living with partner	Partner staying at her parent’s house. Yes = 0, No = 1
Partner’s depression (EPDS)^a^	Ten items measuring symptoms of anxiety and depression in the last 7 days. 8 or Higher score indicate depression. No depression = 0, Depression = 1
Marital relationship satisfaction (QMI)	Six items measuring marital relationship satisfaction. Higher scores indicated higher relationship satisfaction.

^a^Data were collected from mothers using self-complete questionnaires

Norton (1983) developed a 1-dimensional scale of the Quality of Marriage Index (QMI) for evaluating marital relationship satisfaction [23]. This scale includes six questions answered on the following scale: considerably agree (4 points), somewhat agree (3 points), somewhat disagree (2 points), and hardly agree at all (1 point). The total score represents relationship satisfaction. The highest possible score is 24, while the lowest possible score is 6. Higher scores indicated higher relationship satisfaction. Moroi examined the adaptation of the QMI for Japanese mothers whose children went to a nursery school or a kindergarten, and reported its reliability (Good-Poor Analysis: *p* < 0.001, I-T correlation: *r* = 0.721–0.835, first factor loading, 0.802–0.892), and internal consistency (Cronbach’s alpha, 0.927) [24]. Cronbach’s alpha of the QMI for the current samples of fathers was 0.88.

#### Data analysis

Fathers with an EPDS score of 8 points or higher, and mothers with a score of 9 points or higher, were classified as the depression group.

The demographic characteristics of fathers were compared between the presence and absence of depression by unpaired t-test and Fisher’s exact test. In order to clarify the factors related with paternal depression, a logistic regression analysis was conducted with depression as the dependent variable and age, part-time work or unemployment, experience of visiting a medical institutions due to a mental health problem, economic anxiety, unexpected pregnancy, pregnancy with infertility treatment, first child, partner’s depression, and lower marital relationship satisfaction as explanatory variables.

SPSS version 22 was used for statistical analysis and the significance level was set at 0.05.

## Results

**Fig. 1:**
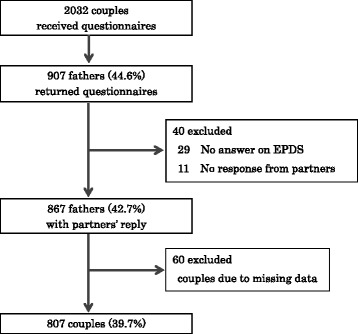
Recruitment process

The recruitment process is shown in Fig. 1. Responses were obtained from 907 fathers (44.6 %), and we analysed data from 807 couples (39.7 %) in which both the father and mother completed all measures. Among the 807 fathers, 110 (13.6 %) had EPDS scores of 8 or above. The mean age of these fathers was 33.4 ± 5.7 years.

Demographics characteristics, health status, psychological factors, and information on pregnancy, childbirth, and engaging with the child are shown in Table 2. Education level was similar to that of the general population. Paternal age and percentage of primiparae were the same as the averages of the general population. Most subjects delivered at term. In contrast, part-time workers and the unemployed were under-represented. There were no significant differences between depressed and non-depressed fathers.

**Table 2:** Comparison of demographic characteristics between depressed and non-depressed fathers

Characteristics mean ± SD or %	With probable depression *n* = 110	Non-depression *n* = 697	*p*
Age (years)	34.4 ± 6.2	33.2 ± 5.6	0.050^a^
University graduate or higher	55.5	54.5	0.918^b^
Part-time work or unemployed	5.5	5.7	1.000^b^
Current illness other than mental disorders	5.5	4.7	0.810^b^
Stressful life events in the previous 1 year	24.5	20.9	0.384^b^
Pregnancy before marriage	13.6	11.2	0.426^b^
First child	57.3	53.4	0.472^b^
Single birth	98.2	99.6	0.140^b^
Gestational week at delivery^c^	39.0 ± 1.5	38.8 ± 1.7	0.251^a^
Children has a health problem^d^	3.7	3.9	1.000^b^

^a^unpaired t-tests, ^b^Fisher’s exact test, ^c^ *n* = 781, ^d^ *n* = 800

Data of general population in Japan

Mean age at the first marriage: 30.8 years [39], Non-regular worker rate: 36.6 % [40],

Overall unemployment rate: 4.3 % [40], University enrollment rate: 56.7 % [41], Primipara rate: 46.7 % [42]

A logistic regression analysis adjusting for all variables in Table 3 was conducted to calculate the odds ratios for paternal postnatal depression. Regarding the relationship with partners, partner’s depression was positively correlated with paternal postnatal depression (adjusted odds ratio (AOR) 1.91; 95 % confidence interval (CI) 1.05–3.47), while marital relationship satisfaction was negatively correlated with it (AOR 0.83; 95 % CI 0.77–0.89). A history of infertility treatment was also significantly correlated with paternal postnatal depression (AOR 2.37; 95 % CI 1.32–4.24). As for psychological factors, experience of visiting a medical institution due to a mental health problem (AOR 4.56; 95 % CI 2.06–10.08) and economic anxiety (AOR 2.15; 95 % CI 1.34–3.45) were significantly correlated with paternal postnatal depression.

**Table 3:** Logistic regression analysis of relevant factors to paternal depression

Variables mean ± SD or %	With probable depression *n* = 110	Non-depression *n* = 697	Crude		Adjusted	
			OR	95 % CI	OR	95 % CI
Age (years)	34.4 ± 6.2	33.2 ± 5.6	1.04	1.00–1.07	1.03	0.99–1.07
Part-time work or unemployed	5.5	5.7	0.95	0.39–2.29	0.79	0.30–2.08
Experience of visiting a medical institution due to a mental health problem	11.8	2.9	4.54	2.19–9.41	4.56	2.06–10.08
Economic anxiety	70.9	49.4	2.50	1.62–3.87	2.15	1.34–3.45
Unexpected pregnancy	4.5	2.3	2.03	0.73–5.65	1.96	0.62–6.17
Pregnancy with infertility treatment	23.6	11.0	2.49	1.51–4.11	2.37	1.32–4.24
First Child	57.3	53.4	1.17	0.78–1.76	1.20	0.75–1.91
Partner’s depression	20.0	8.8	2.61	1.53–4.46	1.91	1.05–3.47
Lower marital relationship satisfaction	19.6 ± 3.5	21.6 ± 2.6	0.80	0.75–0.86	0.83	0.77–0.89

OR: odds ratio, CI: confidence interval

Adjusted ORs were the value after adjusting for all items in the table

## Discussion

This study aimed to clarify the prevalence and the correlated factors of paternal postnatal depression among a large sample of Japanese fathers. Our results showed that the prevalence of paternal depression at four months after childbirth was 13.6 %. The factors that were significantly correlated with paternal depression were the presence of partner’s depression, low marital relationship satisfaction, pregnancy with infertility treatment, experience of visiting a medical institution due to a mental health problem, and economic anxiety.

The prevalence of 13.6 % obtained in the present study is low compared to Paulson’s report at 3–6 months after childbirth (25.6 %) [8]. However, the prevalence of paternal depression increased from 13.6 % during partners’ pregnancy to 16.3 % at 6 months and 19.4 % at 12 months after childbirth in Japan [15]. In a previous report in Portugal, paternal postnatal depression after 12 months postpartum secondarily occurred following maternal depression [25]. The reality of family and social life after childbirth is quite different from parents’ expectations during pregnancy and this discrepancy could be a key predictor of parents’ depression at 3 months after childbirth [26].

Maternal depression was significantly associated with paternal depression (AOR 1.91). In our previous study, paternal depression was not correlated with maternal depression at one month after childbirth [13]. Some Japanese mothers traditionally return to their parents’ home before delivery and stay there for one month after childbirth, suggesting that changes in family and social life have not yet affected their partners at one month after childbirth. Accordingly, fathers in the present study experienced these changes during the four months following childbirth.

Interestingly, low marital relationship satisfaction was correlated with a high prevalence of paternal postnatal depression (AOR 0.83) in this study. In some previous reports, low marital satisfaction was strongly associated with paternal postnatal depression [11], and was related to a mother’s difficulty with child-rearing other than the mother’s depression [12]. Based on these results, an unsatisfactory marital relationship elicited by changes due to childbirth are associated with a partner’s depression.

Furthermore, history of infertility treatment was correlated with paternal postnatal depression (AOR 2.37). In the present study, 103 couples (12.8 %) achieved pregnancy through infertility treatment and this rate was higher than 9.0 % in the international estimates reported by Boivin et al. [27]. Infertility treatments induce anxiety and depression among women and their partners [28, 29], and the influence differs between them [30], suggesting that infertility treatment may affect the relationship between partners. Along with progress in infertility treatments, the chance of postnatal mental health problems may increase in fathers.

The strongest predictor of paternal depression was the experience of visiting a medical institution due to a mental health problem (AOR 4.56). This suggests that regardless of the past history of mental disorders, vulnerability to mental health problems can increase the risk for developing postnatal depression. In our previous study, a similar relationship was also evident when predicting paternal depression at one month after childbirth [13]. These results indicate that it is important for healthcare providers to be mindful of fathers who have previously visited a medical institution due to a mental health problem during the pregnancy period in order to initiate the required support as soon as possible.

Economic anxiety was correlated with paternal depression (AOR 2.15), but employment status was not. Gaillo et al. reported that work environment, e.g., flexible working hours, parental leave, and autonomy over workload, were relative factors persistently associated with paternal depression [31, 32]. In the present study, there were 46 fathers (5.7 %) who were not regularly employed or who were unemployed, and this percentage is smaller than the rate of non-regular employment within Japan in 2012 (19.8 %) [33]. Only 6 fathers in the non-regular employment or unemployment group were depressed and this cell number was insufficient for a logistic regression analysis. In our previous study of Japanese fathers at one month postpartum, employment status was strongly correlated with paternal postnatal depression [13]. To confirm the association between paternal postnatal depression and work environment in Japan, we need to recruit more non-regularly employed and unemployed fathers.

In conclusion, it is important for healthcare providers to understand the mental health status of both fathers and mothers at four months after childbirth. When dealing with maternal depression, it is important to consider the possibility that fathers are not able to fully provide support to mothers and might also need some support. In Japan, there are reports of delayed action regarding perinatal mental health in regional central hospitals that receive pregnant women with psychiatric problems [34]. Since proper child-rearing is a social responsibility, it is necessary to build better support systems that provide aid to both mothers and fathers after childbirth.

### Limitations

Some limitations of the current study should be mentioned. The first limitation is the study design. In the cross-sectional design, it is difficult to clarify causal relationships among factors correlated with paternal postnatal depression. Additionally, questionnaires for mothers and fathers were sent to all mothers 4 months after childbirth along with guidance. In the document, we instructed mothers and fathers to not discuss their responses together. However, as they completed their questionnaires at home, we could not know whether mothers and fathers really did not discuss this. In the next study, we should send the questionnaires for fathers and mothers separately instead of sending them both to the mothers.

Second, the response rate was insufficient and the representativeness of the sample was problematic. The response rates from fathers and mothers were 44.6 and 51.0 %, respectively. However, the response rate from partners in a couple was only 39.7 %, which was due to the lower response rate of fathers. Generally, the response rates of fathers in similar studies have been very low, so a low response rate was expected before the study [35]. To improve the response rate of fathers, a direct request for fathers to participate in the study is needed.

Third, we did not use a diagnostic interview to assess depression. The EPDS is a common scale used to assess postpartum depression, especially in large-scale studies [1, 2]. Accordingly, the findings of the study should be limited and applied to fathers with probable depression as assessed by the EPDS.

Fourth, the results of this study should not be generalized due to the sample bias, especially the low rate of fathers with unstable employment status. Statistical bias of couple’s EPDS scores also causes difficulty in generalization because these scores are not independent. In a future study, we need to use probability sampling from the broader population and conduct couple/dyadic analysis where an assumption of independence is not made [36].

## Conclusions

This was the first report of the relevant factors of paternal postnatal depression at four months postpartum in Japan, including the relationship with partners. The prevalence of paternal depression at four months after childbirth was 13.6 % in Japan. Postnatal depression among Japanese fathers was correlated with maternal depression at four months after childbirth, although it was not correlated at one month after childbirth, as reported in our previous study [13]. Moreover, these depressed fathers had poor relationship satisfaction with their partners. Some Japanese mothers stay at their parents’ home for their childbirth, and return to their home to live together with the father and their newborn from one month postpartum, suggesting that their mental condition, e.g., depression and poor marital satisfaction, had a mutual influence after starting to live together.

Currently, in Japan, 1-month and 4-month infant health examinations are provided for all families, and midwives or public health nurses visit all families within four months after childbirth. In a suspected case of maternal depression, trained public health nurses usually perform two or more home-visits and confirm depressive symptoms by interviews, e.g., Mini-International Neuropsychiatric Interview [37], and refer subjects with marked depressive symptoms to psychiatrists.

In the future, health providers will need to use EPDS screening for both mothers and fathers. We are planning to develop a new health condition questionnaire including the Quality of Marriage Index (QMI) for evaluating marital satisfaction and the simple screening scale of domestic violence, e.g., Partner Violence Screening (PVS) [24, 38]. We will provide EPDS/QMI for fathers and EPDS/QMI/PVS for mothers. The father and mother will have a separate questionnaire to complete and place in a sealed envelope in order to prevent them seeing from each other’s responses. Two separate questionnaires will be handed to mothers at the 1-month check. The father and mother will complete their respective questionnaires, put them in separate envelopes, and seal the envelopes, individually. At the time of the 4-month home-visit, we will recover the two questionnaires from the mother.

## Abbreviation

EPDS: The Edinburgh Postnatal Depression Scale
